# Beneficial effects of end-ischemic oxygenated machine perfusion preservation for split-liver transplantation in recovering graft function and reducing ischemia–reperfusion injury

**DOI:** 10.1038/s41598-021-01467-0

**Published:** 2021-11-19

**Authors:** Daisuke Ishii, Naoto Matsuno, Mikako Gochi, Hiroyoshi Iwata, Tatsuya Shonaka, Yuji Nishikawa, Hiromichi Obara, Hideki Yokoo, Hiroyuki Furukawa

**Affiliations:** 1 Department of Surgery, Asahikawa Medical University, 2-1-1-1 Midorigaoka-Higashi, Asahikawa, Hokkaido 078-8510 Japan; 2 Department of Pathology, Asahikawa Medical University, Asahikawa, Japan; 3 Department of Mechanical Engineering, Tokyo Metropolitan University, Tokyo, Japan

**Keywords:** Preclinical research, Liver diseases

## Abstract

This study examined the efficacy of end-ischemic hypothermic oxygenated machine perfusion preservation (HOPE) using an originally developed machine perfusion system for split-liver transplantation. Porcine split-liver grafts were created via 75% liver resection after 10 min of warm ischemia. In Group 1, grafts were preserved by simple cold storage (CS) for 8 h (CS group; n = 4). In Group 2, grafts were preserved by simple CS for 6 h and end-ischemic HOPE for 2 h (HOPE group; n = 5). All grafts were evaluated using an isolated ex vivo reperfusion model with autologous blood for 2 h. Biochemical markers (aspartate aminotransferase and lactate dehydrogenase levels) were significantly better immediately after reperfusion in the HOPE group than in the CS group. Furthermore, the HOPE group had a better histological score. The levels of inflammatory cytokines (tumor necrosis factor-α, interferon-γ, interleukin-1β, and interleukin-10) were significantly lower after reperfusion in the HOPE group. Therefore, we concluded that end-ischemic HOPE for split-liver transplantation can aid in recovering the graft function and reducing ischemia–reperfusion injury. HOPE, using our originally developed machine perfusion system, is safe and can improve graft function while attenuating liver injury due to preservation.

## Introduction

With the increasing number of patients waiting for liver transplantation, donor shortage has become a serious problem. Split-liver transplantation can help increase the available donor pool but can create two extended-criteria grafts and increase the risk of transplant failure^1,2^. Hence, split-liver grafts may be considered marginal because of their small size^3^ and the degree of incurred injury due to liver splitting^4^. Moreover, poor post-transplantation outcomes have been reported even with a warm ischemic time of 10 min^5^. We reported higher liver deviation enzyme levels and vascular resistance during reperfusion with split-liver grafts than those with whole-liver grafts^6^. Our experiments in pigs demonstrated that the split-liver graft is a marginal graft and cold storage (CS) is unsuitable for storing these grafts^6^. The use of marginal donor livers is associated with a high risk of primary graft dysfunction or severe ischemic injury and can lead to poor outcomes, thus requiring machine perfusion (MP)^7^. In MP, oxygen, electrolytes, and nutritional elements are continuously provided and harmful metabolites are washed out. Therefore, MP can help maintain, recover, and evaluate graft functions.

Over the past decade, end-ischemic perfusion after CS has become a more attractive option than simple CS^8–11^. End-ischemic perfusion can be performed after organ transport for pre-transplant evaluation, and we consider it to be the most clinically relevant method for improving graft function after preservation. It implies a lower risk of shear stress because of a shorter perfusion time^12^. The idea behind end-ischemic MP after initial CS is based on the assumption that metabolic and structural changes during the ischemic period may not be irreversible. In fact, in contrast to exposure of any ischemic tissue to oxygen under normothermic conditions, oxygen delivery under cold conditions has been reported to be very effective in increasing cellular energy with only minor oxidative stress^13^. The underlying mechanism predominantly involves mitochondrial repair^14,15^. End-ischemic hypothermic oxygenated perfusion considerably increases the levels of adenosine triphosphate (ATP) in several tissues^16,17^ and decreases the levels of reactive oxygen species (ROS) and damage-associated molecular patterns (DAMPs) subsequently released during implantation^13,14,18^.

In collaboration with an industrial machinery company, we developed the first proprietary hospital-based perfusion storage device for livers in Japan. This device is ideal for end-ischemic hypothermic oxygenated machine perfusion preservation (HOPE).

To our knowledge, this is the first study on HOPE for split-liver transplantation in pigs. Here, we evaluated the effectiveness and mechanism of end-ischemic HOPE using our MP system for split-liver transplantation.

## Methods

### Animals

For this study, 2–3-month-old female domestic pigs weighing approximately 20 kg (Large-Yorkshire, Landrace, and Duroc crossbred hogs; Taisetsu-Sanroku-Sya, Asahikawa, Japan) were used. The pigs were brought into a gauge equipped with an automatic water supply system 3–4 days before the experiment and fed with 1.2–1.5 kg of food daily. Additionally, they were housed in a temperature- and humidity-controlled environment (12-h light/12-h dark cycle).

This study is reported in accordance with the ARRIVE guidelines for reporting experiments involving animals. All animals received humane care following the Guide for the Care and Use of Laboratory Animals (National Institutes of Health publication 86-23, revised in 1985). The Institutional Animal Ethics Committee of the Clinical Research Center at Asahikawa Medical University, Japan (permit no. 14172) approved all experimental procedures.

### Liver procurement

1. Fasting management was performed 12 h before the experiment for safe administration of general anesthesia.
2. Midazolam (0.25 mg/kg), medetomidine hydrochloride (0.05 mg/kg), and butorphanol tartrate (0.25 mg/kg) were injected for anesthetic induction.
3. A peripheral ear vein was cannulated with a 22G intravenous catheter, and Ringer’s lactate solution was infused at a rate of 200 mL/h.
4. Thiamylal sodium (15 mg/kg) was intravenously injected, and intubation was performed using a 5.5-mm endotracheal tube.
5. Anesthesia was maintained with isoflurane (2%) (Forane VR; Abbot Japan, Tokyo, Japan) and oxygen (2 L/min). The respiratory conditions were as follows: tidal volume, 10–15 mL/kg; respiratory rate, 18 breaths/min; and inspiratory:expiratory ratio, 1:2. All experiments were performed under sufficient anesthesia.
6. A 6-Fr central venous catheter was inserted through the left internal jugular vein, and 500 mL of 6% hydroxyethyl starch (Fresenius Kabi, Bad Homburg, Germany) was infused until the end of autologous blood procurement.
7. The domestic pigs underwent midline laparotomy.
8. The right common iliac artery was isolated, and a 14-Fr Nelaton catheter was inserted into the vein to procure approximately 1000 mL of autologous whole blood.
9. In preparation for liver exposure, the hepatic artery (HA), portal vein (PV), bile duct, and upper and lower parts of the hepatic inferior vena cava were dissected and taped.
10. The abdominal aorta was cross-clamped to start warm ischemia. Simultaneously, cardiac arrest was induced with intravenous potassium chloride (2 mEq/kg). No heparin was administered before cardiac arrest.
11. After 10 min (10 min of warm ischemic time simulated the donation after circulatory death (DCD) Liver), 1000 mL of Euro-Collins solution containing 3000 units of heparin at 4 °C was perfused from the right common iliac artery. While cooling the liver with crushed ice, the liver was rapidly removed.
12. Standard bench preparation was performed. The portal and arterial systems were cannulated using our original plastic perfusion cannulas of appropriate size. The isolated liver was washed with 1000 mL of UW-gluconate solution at 4 °C.
13. Porcine split-liver grafts were created via 75% liver resection.
14. Grafts were preserved by CS in UW-gluconate solution at 4 °C.

### Perfusion preservation machine

**Figure 1:**
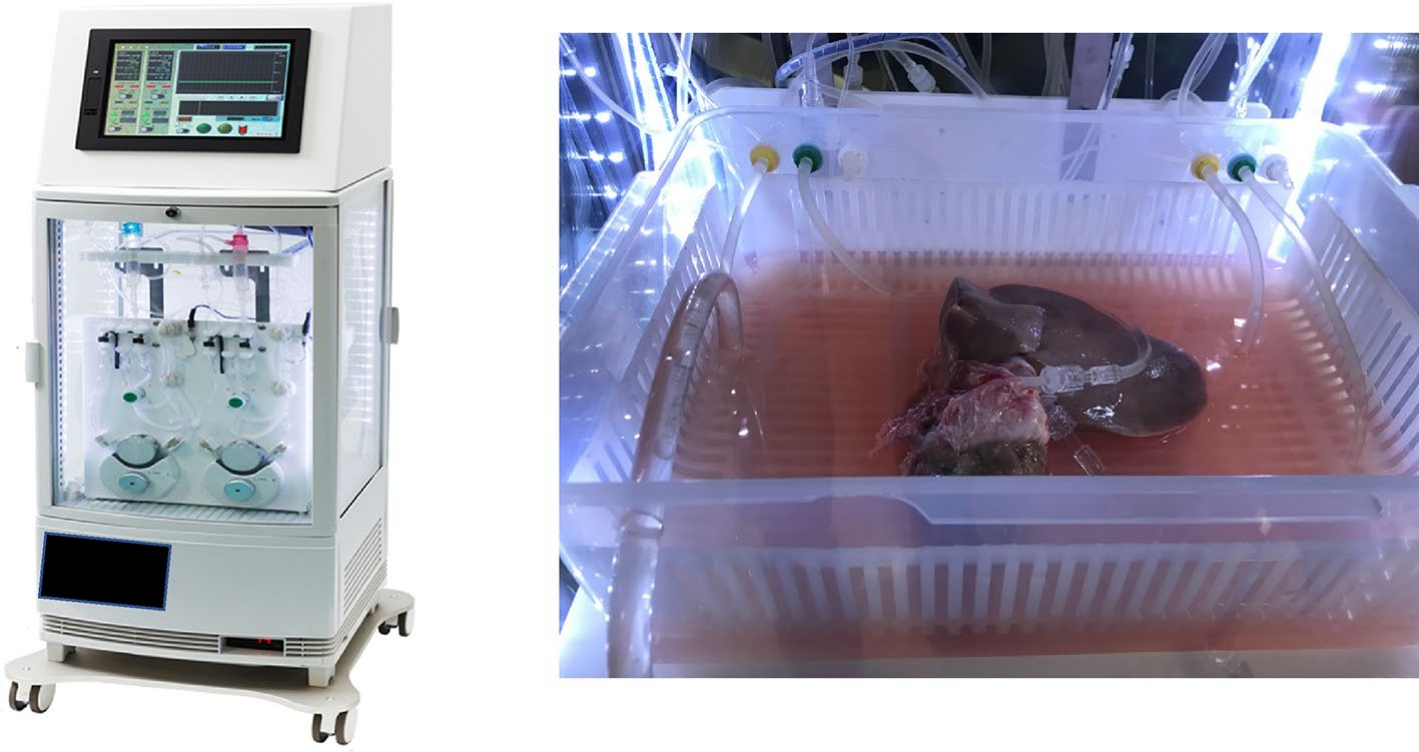
Perfusion preservation machine. The grafts were perfused using an originally developed MP system (CMP-X04W; Chuo Seiko Co., Ltd., Asahikawa, Japan).

The grafts were perfused using an originally developed MP system (CMP-X04W; Chuo Seiko Co., Ltd., Asahikawa, Japan) (Fig. 1). The perfusion pressure was controlled at 5–8 and 30–50 mmHg for the PV and HA, respectively, by adjusting the flow rate. The perfusion fluid was a UW solution, with the temperature set at 4 °C. The partial oxygen pressure in the perfusate was controlled at 250 mmHg (FiO_2_ of 0.4 at 2 L/min). The perfusion time was set at 2 h after 6 h of simple CS.

### Experimental design

**Figure 2:**
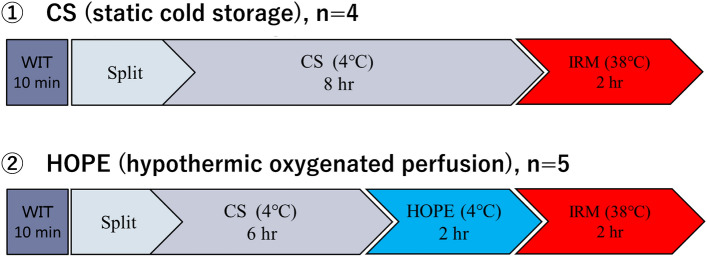
Experimental design. Porcine split-liver grafts were created via 75% liver resection after 10 min of warm ischemia. In Group 1, grafts were preserved by simple CS for 8 h (CS group; n = 4). In Group 2, grafts were preserved by simple CS for 6 h and end-ischemic HOPE for 2 h (HOPE group; n = 5). All grafts were evaluated using an isolated ERM with autologous blood for 2 h. *CS* cold storage, *ERM* ex vivo reperfusion model, *HOPE* hypothermic oxygenated machine perfusion preservation.

Figure 2 illustrates the experimental design. Porcine split-liver grafts were created via 75% liver resection after 10 min of warm ischemia. In Group 1, grafts were preserved by simple CS for 8 h (CS group; n = 4). In Group 2, grafts were preserved by simple CS for 6 h and end-ischemic HOPE for 2 h (HOPE group; n = 5). All grafts were evaluated using an isolated ex vivo reperfusion model (ERM) with autologous blood for 2 h.

### Isolated ERM

After preservation, the liver function was evaluated using an isolated liver reperfusion model^6,19,20^. This system consisted of HA and PV perfusion circuits, with each circuit comprising a roller-type pump (Masterflex 7520-40; Cole-Parmer, Bunker Court, IL, USA), electrical flow meter (VN05; Aichi Tokei, Aichi, Japan for the PV; FD-SS02; Keyence, Osaka, Japan for the HA), ceramic capacitive pressure sensor (KL76; Nagano Keiki, Nagano, Japan), and an air trap developed in-house. An oxygenator (HP0-06 H-C; Senko Medical Instrument, Tokyo, Japan) was installed in the circuit for the PV and HA.

After preservation, all the organs were rinsed with 500 mL of cold Euro-Collins solution and subsequently exposed at room temperature (20–25 °C) without perfusion to simulate the slow rewarming of grafts during surgical implantation in vivo. HA reperfusion was set at 80 mmHg and automatically maintained by a roller pump connected to a pressure sensor placed in the inflow line immediately before the arterial cannula. PV reperfusion was set at 5–8 mmHg. The reperfusion fluid was autologous whole blood containing 30 mL of calcium gluconate hydrate (8.5%) and heparin. Heparin levels were adjusted for the activated clotting time to be 300 s. The livers were subsequently reperfused with oxygenated diluted autologous blood at 38 °C. The hematocrit was maintained at approximately 10–12%. The oxygenator was regulated to achieve physiological blood gas values (pO_2_, approximately 150–200 mmHg; pCO_2_, approximately 30–50 mmHg).

### Viability assessment during MP

Aspartate aminotransferase (AST), lactate dehydrogenase (LDH), alkaline phosphatase, and hyaluronic acid levels in the perfusate were measured at 0 and 2 h for HOPE and every 60 min for the ERM to determine the viability of preserved liver grafts using standard biochemical methods (clinical grade). Additionally, lactate, potassium, and carboxyhemoglobin (CO-Hb) levels in the perfusate were measured every 60 min for the ERM using a blood gas analyzer (ABL800 FLEX; Radiometer, Tokyo, Japan).

### Histopathological evaluation

Liver wedge biopsy was performed immediately after laparotomy and preservation (simple CS and HOPE) and 2 h after isolated ex vivo reperfusion. Tissue ATP levels were quantified using homogenized liver biopsy tissues. Liver tissues were fixed using 10% phosphate-buffered formalin and dehydrated using ethanol. Paraffin-embedded renal sections (4 μm) were stained with hematoxylin and eosin for morphological evaluation. The Suzuki classification^21^ was applied to evaluate ischemia–reperfusion injury (IRI). Sinusoidal congestion, hepatocyte cytoplasmic vacuolization, and parenchymal necrosis were scored from 0 to 4. The histopathological findings of each hepatic section were scored blindly in ≥ 10 randomly selected non-overlapping fields under light microscopy.

CD42b and ERG immunohistochemistry was performed to assess sinusoidal endothelial cell injury and intrasinusoidal platelet aggregation. The primary antibodies were a rabbit anti-ERG monoclonal antibody (clone EP111; Nichirei Bioscience, Tokyo, Japan) and a rabbit anti-CD42b polyclonal antibody (GeneID 2811; Proteintech Group, IL, USA). The secondary antibody was an EnVision-labeled polymer reagent (Dako, Glostrup, Denmark). Anti-ERG staining-positive and anti-CD42b staining-positive areas were quantified using ImageJ (US National Institute of Health, Bethesda, MD, USA). The number of anti-ERG staining-positive sinusoid epithelial cells (SEC) nuclei and the positive area of anti-CD42b staining were automatically counted in ≥ 10 randomly selected non-overlapping fields.

### Gene expression

Quantitative reverse transcription-polymerase chain reaction was used to determine gene expression of endothelial-specific, inflammatory, and apoptotic proteins. Liver parenchyma biopsy was performed after laparotomy and preservation and 2 h after isolated ex vivo reperfusion. The samples were stored at − 80 °C until analysis. Total RNA was extracted from snap-frozen liver biopsies using the miRNeasy Micro Kit (Qiagen, Valencia, CA, USA). The RNA concentration was determined using a spectrophotometer (NanoDrop 2000; NanoDrop Technologies, Wilmington, DE, USA). Equal RNA amounts were converted to complementary DNA using the Transcriptor First Strand cDNA Synthesis Kit (Roche, Basel, Switzerland); complementary DNA levels were measured using LightCycler 480 System II (Roche, Basel, Switzerland). The relative expression of the mRNA of interest was normalized to the housekeeping gene glyceraldehyde-3-phosphate dehydrogenase (GAPDH). The data are presented as relative quantification to GAPDH. Table 1 summarizes sense and antisense primer sequences.

**Table 1:** PCR primer.

Primer	Forward	Reverse
GAPDH	AGGAGTAAGAGCCCCTGGAC	GTGTGTTGGGGGATCGAGT
TNF-α	TTGTCGCTACATCGCTGAAC	CCAGTAGGGCGGTTACAGAC
IFNγ	TTCAGCTTTGCGTGACTTTG	TGCATTAAAATAGTCCTTTAGGATCG
IL-1β	GGAAAGCCATACCCAGAGGT	CAGTCCCCTTCTGTCAGCTT
Caspase-3	GAATGGCATGTCGATCTGGT	TTGTGAAGGTCTCCCTGAGATT
NOS2	CCATGGAACACCCCAAATAC	GCAGGGCGTACCACTTCA
IL-4	GAGAACACGACGGAGAAGGA	TCTGTAGATGTGCCGAAGCA
IL-6	TGAACTCCCTCTCCACAAGC	GGCAGTAGCCATCACCAGA
IL-10	TCCAGTTTTACCTGGAAGACG	CCTTGATATCCTCCCCATCA

### Statistical analyses

All statistical analyses were performed using EZR version 1.41 (Saitama Medical Center, Jichi Medical University, Saitama, Japan)^22^, based on R and R commander. The results were obtained using the t-test. *P*-values < 0.05 were considered statistically significant.

## Results

### HOPE results

The perfusion volumes of both portal and hepatic arteries were stabilized at 0.50 and 0.05 mL/min/100 g liver, respectively, within 15 min after starting perfusion preservation and maintained until the end of perfusion preservation. The storage vessel temperature during perfusion preservation was stable at 4 °C. The AST and LDH levels were not significantly different at the start and end of perfusion preservation. The AST levels at 0 and 120 min of perfusion preservation were 82.33 ± 14.13 and 115.48 ± 27.60 IU/L/100 g liver, respectively (0 vs. 120 min; *P* = 0.316), whereas LDH levels at 0 and 120 min of perfusion preservation were 88.22 ± 0.87 and 145.42 ± 11.37 IU/L/100 g liver, respectively (0 vs. 120 min; *P* = 0.056).

### Liver enzyme levels after reperfusion and IRI

#### AST levels in the perfusion solution

**Figure 3:**
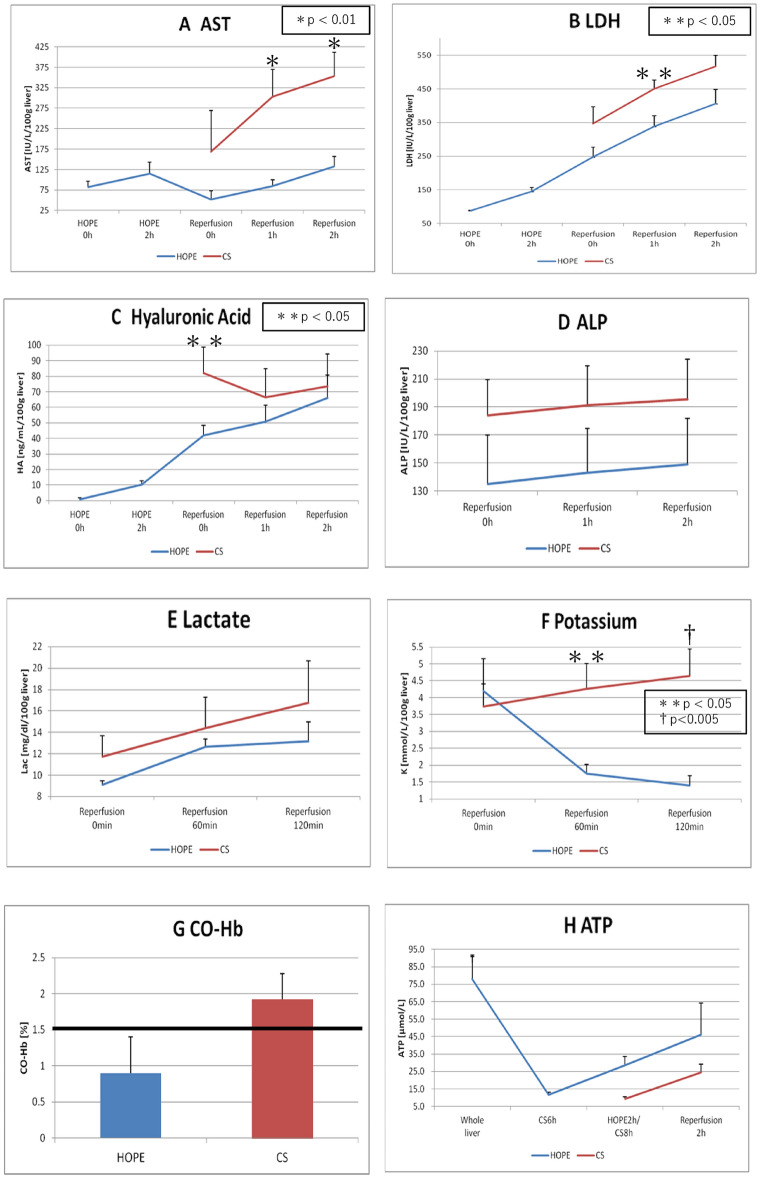
Laboratorial results. (**A**) Aspartate aminotransferase concentration (IU/L/100 g liver), (**B**) lactate dehydrogenase concentration (IU/L/100 g liver), (**C**) hyaluronic acid concentration (ng/mL/100 g liver), (**D**) alkaline phosphatase concentration (IU/L/100 g liver), (**E**) lactate concentration (mg/dL/100gliver), and (**F**) potassium concentration (mmol/L/100 g liver) value (mean ± SD) in the perfusate over time (hours) during HOPE and ERM. (**G**) Carboxyhemoglobin concentration (%) values (mean ± SD) in the perfusate at 2 h after ERM. (H) Tissue adenosine triphosphate level (μmol/L) values (mean ± SD) after laparotomy, after preservation (simple cold storage [CS] and HOPE), and at 2 h after isolated ERM. *ERM* ex vivo reperfusion model, *HOPE* hypothermic oxygenated machine perfusion preservation, *SD* standard deviation.

Figure 3A presents AST levels after reperfusion. At 60 min and the end of reperfusion, the AST levels were significantly lower in the HOPE group (84.25 ± 15.90 and 132.31 ± 24.80 IU/L/100 g liver) than in the CS group (303.40 ± 66.75 and 353.12 ± 58.94 IU/L/100 g liver) (HOPE vs. CS; *P* = 0.009, *P* = 0.007).

#### LDH levels in the perfusion solution

Figure 3B presents LDH levels after reperfusion. At 60 min of reperfusion, the LDH levels were significantly lower in the HOPE group than in the CS group (338.43 ± 31.75 vs. 450.86 ± 25.36 IU/L/100 g liver; *P* = 0.033). The LDH levels at the end of reperfusion were not different between the groups (406.36 ± 41.50 IU/L/100 g liver [HOPE] vs. 516.61 ± 32.84 IU/L/100 g liver [CS]; *P* = 0.086).

#### Hyaluronic acid levels in the perfusion solution

Figure 3C presents hyaluronic acid levels after reperfusion. At 0 min of reperfusion, the hyaluronic acid levels were significantly lower in the HOPE group than in the CS group (41.88 ± 6.57 vs. 82.20 ± 16.52 ng/mL/100 g liver; *P* = 0.043). However, the hyaluronic acid levels at the end of reperfusion were not different between the groups (66.17 ± 14.64 vs. 73.64 ± 20.73 ng/mL/100 g liver; *P* = 0.771).

#### Alkaline phosphatase levels in the perfusion solution

Figure 3D presents alkaline phosphatase levels after reperfusion; no differences were observed between the groups. At 120 min of reperfusion, alkaline phosphatase levels in the HOPE and CS groups were 149.18 ± 32.87 and 195.31 ± 28.69 IU/L/100 g liver, respectively (*P* = 0.339).

#### Lactate levels in the perfusion solution

Figure 3E presents lactate levels after reperfusion; no differences were observed between the groups. At 120 min of reperfusion, the lactate levels in the HOPE and CS groups were 13.16 ± 1.79 and 16.75 ± 3.92 mg/dL/100 g liver, respectively (*P* = 0.399).

#### Potassium levels in the perfusion solution

Figure 3F presents potassium levels after reperfusion. At 60 min and the end of reperfusion, the potassium levels were significantly lower in the HOPE group (1.74 ± 0.28 and 1.40 ± 0.27 mmol/L/100 g liver) than in the CS group (4.26 ± 0.74 and 4.64 ± 0.80 mmol/L/100 g liver) (HOPE vs. CS; *P* = 0.010, *P* = 0.004).

#### CO-Hb levels in the perfusion solution

Figure 3G presents CO-Hb levels at the end of reperfusion. The CO-Hb level, an IRI marker, was > 1.5% in the CS group. However, CO-Hb levels at the end of reperfusion were not different between the groups (0.90 ± 0.50% vs. 1.93 ± 0.36%; *P* = 0.159).

#### ATP levels in liver tissues

Figure 3H presents ATP levels in liver tissues. At the end of reperfusion, ATP levels were not different between the groups (46.20 ± 40.30 vs. 24.48 ± 9.72 µmol/L; *P* = 0.333).

### Vascular flow volume and pressure resistance

#### PV flow and pressure resistance after reperfusion

**Figure 4:**
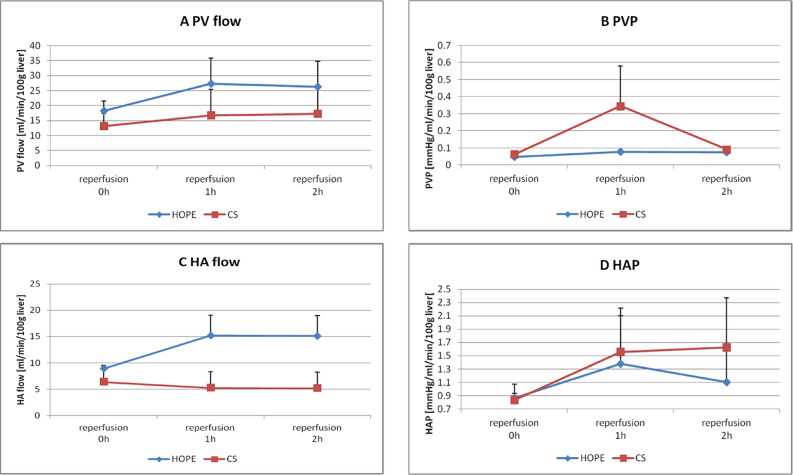
Vascular flow volume and pressure resistance. (**A**) Portal vein flow (mL/min/100 g liver), (**B**) PV pressure (mmHg/mL/min/100 g liver), (**C**) hepatic artery flow (mL/min/100 g liver) and (**D**) HA pressure value (mean ± standard deviation) over time (hours) during the ex vivo reperfusion model.

Figure 4A,B present the results for PV flow and pressure, respectively. After reperfusion, no differences in the PV flow and pressure were observed between the groups. At 120 min of reperfusion, the PV flow in the HOPE and CS groups was 26.28 ± 8.46 and 17.22 ± 8.91 mL/min/100 g liver, respectively (*P* = 0.489). At 120 min of reperfusion, the PV pressure in the HOPE and CS groups was 0.07 ± 0.013 and 0.09 ± 0.02 mmHg/mL/min/100 g liver, respectively (*P* = 0.454).

#### HA flow and pressure resistance

Figure 4C,D present the results for HA flow and pressure, respectively. After reperfusion, HA flow and pressure were not different between the groups. At 120 min of reperfusion, HA flow in the HOPE and CS groups was 15.13 ± 3.85 and 5.18 ± 3.04 mL/min/100 g liver, respectively (*P* = 0.125). At 120 min of reperfusion, HA pressure in the HOPE and CS groups was 1.10 ± 0.56 and 1.62 ± 0.75 mmHg/mL/min/100 g liver, respectively (*P* = 0.617).

### Histological findings after reperfusion

#### Hematoxylin–eosin staining

**Figure 5:**
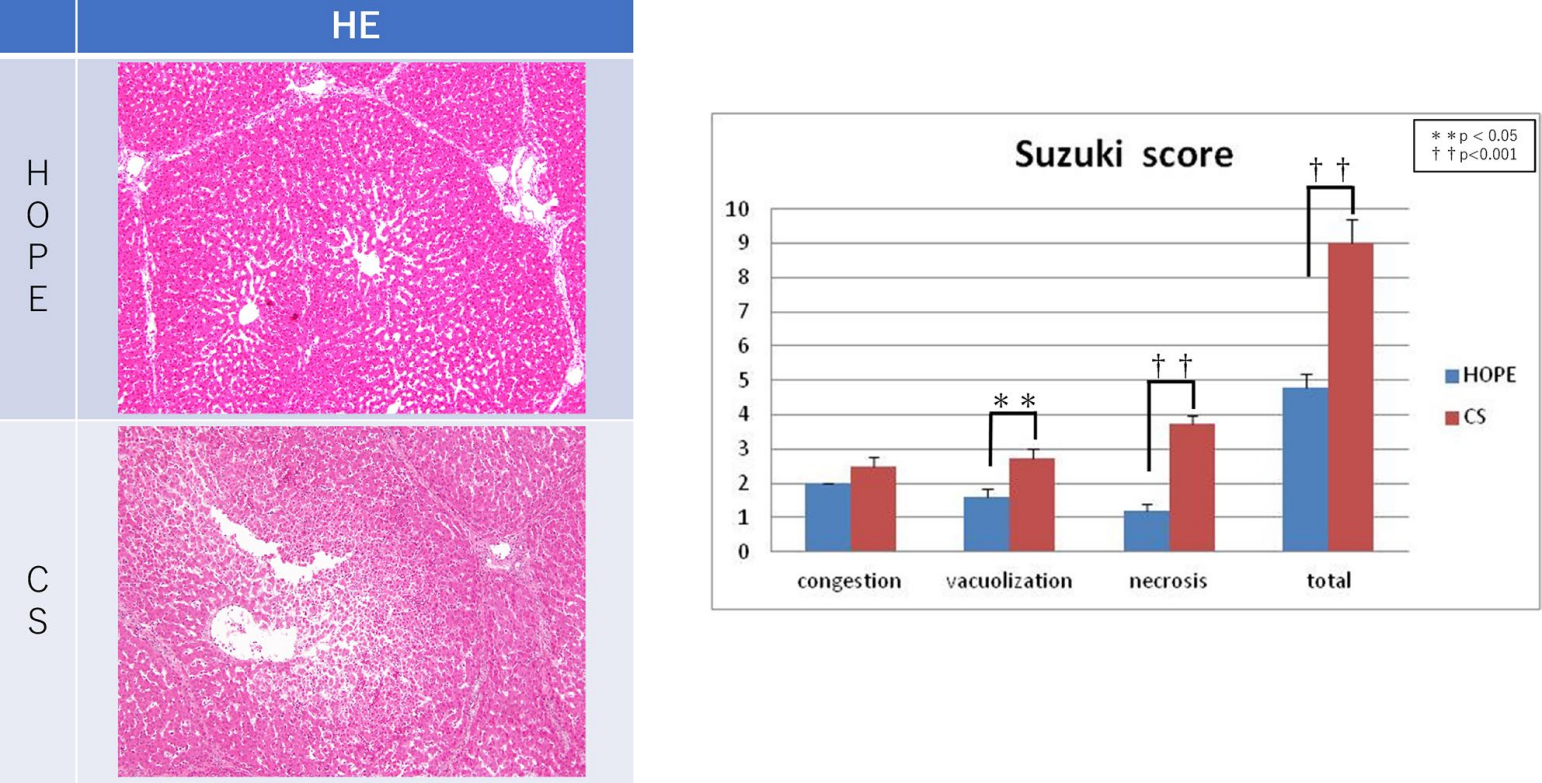
Pathological findings. Histological findings indicated that the liver cell structure was irregular in the CS group but remained regular in the HOPE group. In particular, the sinusoidal structure was maintained in the HOPE group, and more necrotic areas were observed in the CS group. Under high-power magnification, the liver cells exhibited more lipid drops in the CS group than in the HOPE group. The total Suzuki scores indicated significant morphological changes in the HOPE group (4.80 ± 0.37), as compared to the CS group (9.00 ± 0.71) (*P* < 0.001). *CS* cold storage, *HOPE* hypothermic oxygenated machine perfusion preservation.

The histological findings indicated irregular liver cell structure in the CS group but not in the HOPE group (Fig. 5). Specifically, the sinusoidal structure was maintained in the HOPE group and more necrotic areas were observed in the CS group. Under high-power magnification, the liver cells exhibited more lipid drops in the CS group. Figure 5 presents the Suzuki scores for hepatic IRI at the end of reperfusion. The HOPE group scored significantly better regarding vacuolization and necrosis than the CS group. The total Suzuki scores indicated significant morphological changes in the HOPE group (4.80 ± 0.37) compared to the CS group (9.00 ± 0.71) (*P* < 0.001).

#### ERG and CD42b immunohistochemistry

**Figure 6:**
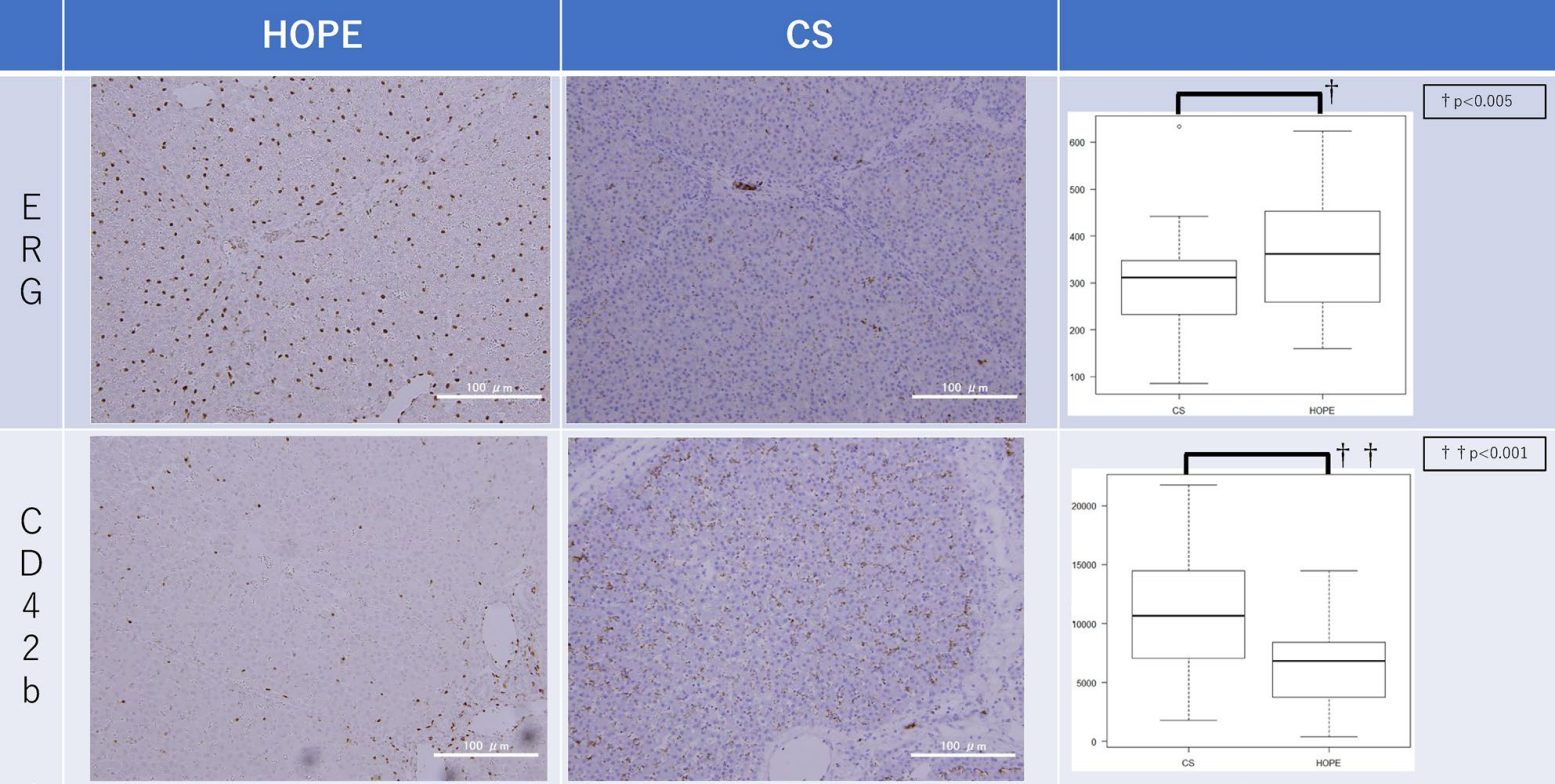
Immunohistochemistry. At the end of reperfusion, the number of anti-ERG staining-positive SEC nuclei counted automatically by ImageJ in the HOPE group (360.54 ± 118.87 per field) was significantly higher than that in the CS group (285.13 ± 107.87 per field) (*P* = 0.002). At the end of reperfusion, the positive area of anti-CD42b staining counted automatically by ImageJ in the HOPE group (6317.06 ± 3235.14 per field) was significantly smaller than that in the CS group (10,761.50 ± 5643.620 per field) (*P* < 0.001). *CS* cold storage, *HOPE* hypothermic oxygenated machine perfusion preservation, *SEC* sinusoid epithelial cells.

Figure 6 shows the ERG and CD42b results. At the end of reperfusion, the HOPE group had significantly higher numbers of anti-ERG staining-positive SEC nuclei than the CS group (360.54 ± 118.87 vs. 285.13 ± 107.87 per field; *P* = 0.002) and a significantly smaller positive area of anti-CD42b staining than the CS group (6317.06 ± 3235.14 vs. 10,761.50 ± 5643.620 per field; *P* < 0.001).

### Gene expression

**Figure 7:**
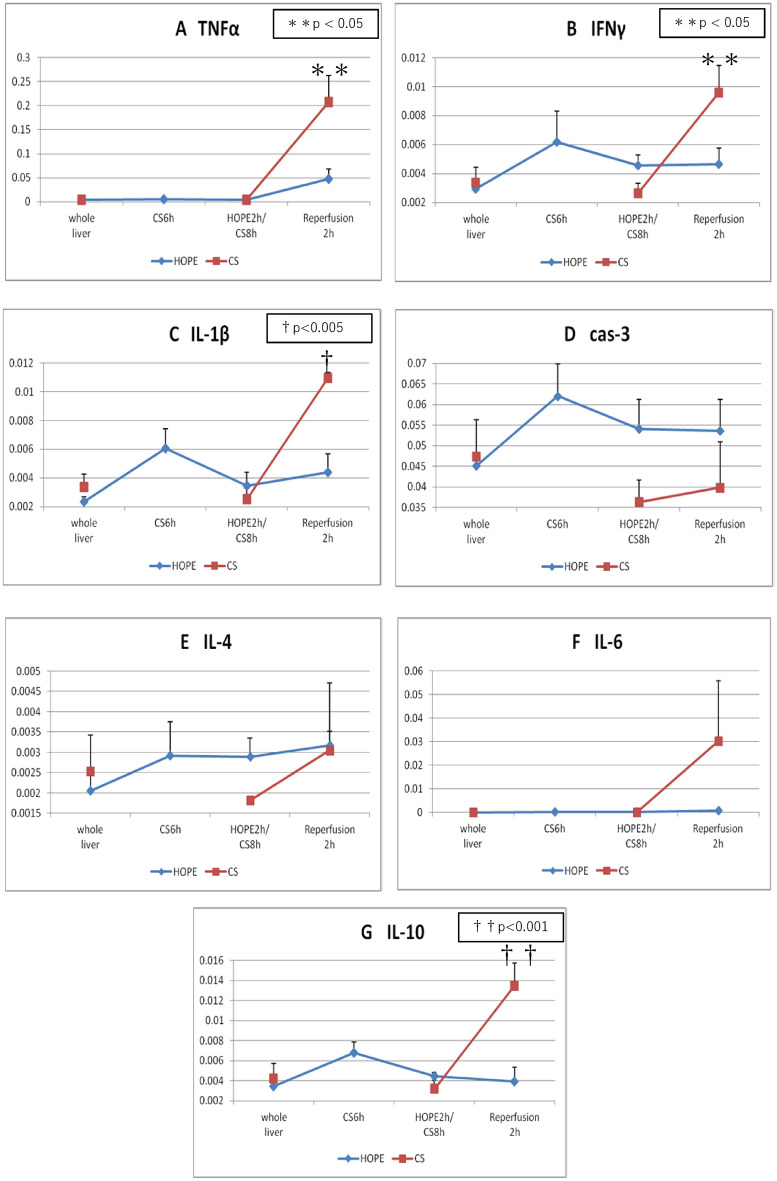
Polymerase chain reaction. (**A**) Tumor necrosis factor α, (**B**) interferon γ, (**C**) IL-1β, (**D**) caspase-3, (**E**) Il-4, (**F**) IL-6 and (**G**) IL-10 value (mean ± SD) after laparotomy, after preservation (simple CS and HOPE), and at 2 h after isolated ERM. *CS* cold storage, *ERM* ex vivo reperfusion model, *HOPE* hypothermic oxygenated machine perfusion preservation, *IL* interleukin, *SD* standard deviation.

Figure 7 shows the gene expression results. At the end of reperfusion, the DAMP gene expression was significantly lower in the HOPE group than in the CS group: tumor necrosis factor (TNF-α), 0.0476 ± 0.0446 vs. 0.2080 ± 0.1084, respectively, *P* = 0.019 (Fig. 7A); interferon-γ, 0.0047 ± 0.0025 vs. 0.0096 ± 0.0038, respectively, *P* = 0.049 (Fig. 7B); interleukin-1β, 0.0044 ± 0.0029 vs. 0.0110 ± 0.0008, respectively, *P* = 0.003 (Fig. 7C); and interleukin-10, 0.0039 ± 0.0032 vs. 0.0135 ± 0.0045, respectively, *P* = 0.008 (Fig. 7G). The HOPE and CS groups did not differ regarding caspase-3 (0.0537 ± 0.0170 vs. 0.0399 ± 0.0221; *P* = 0.323; Fig. 7D), interleukin-4 (0.0032 ± 0.00342 vs. 0.0030 ± 0.0009; *P* = 0.944; Fig. 7E), and interleukin-6 (0.0004 ± 0.0007 vs. 0.0030 ± 0.0512; *P* = 0.228; Fig. 7F) expression.

## Discussion

The optimal temperature and timing for perfusion preservation remain controversial. Recently, there have been several reports on HOPE^13,23–26^. We focused on this preservation method, as we believe it has real clinical relevance. Patrono et al.^23^ reported that the use of HOPE for grafts obtained from donation after brain death was associated with a significant reduction in stage 2–3 acute kidney injury and severe post-reperfusion syndrome, a lower incidence of early allograft dysfunction, and a reduced post-transplantation AST and ALT peak. Although normothermic MP (NMP) has been previously reported^27^, NMP has the following problems. Higher preservation temperatures require higher oxygen demand of the organs^28^. Therefore, dissolved oxygen alone is insufficient in NMP, and oxygen carriers are required. Additionally, metabolism is increased; hence, a dialysis system is also needed to remove waste products, making it more complicated and costly. In NMP, if the perfusion system fails, oxygenation and waste removal may not be possible and organ damage may occur. Conversely, with HOPE, if a failure occurs in the perfusion system, the system simply converts to simple CS, ensuring safety. Furthermore, NMP carries the risk of bacterial contamination, bacterial growth, and coagulation in the circuit^29^.

### Endothelial cell effects

HOPE has at least four protective mechanisms against lethal impairment of hepatic flow in parenchymal and nonparenchymal cells^29^. Flow cessation results in a significant reduction in several endothelial vasoprotective pathways. The negative effects of CS conditions are partly attributable to the loss of Kruppel-like factor 2 expression, a vasoprotective transcription factor^30^. MP may trigger endothelial protection via the upregulation of shear stress-sensitive protective genes^31^. Our ERG immunohistochemistry experiments showed that the HOPE group had significantly more endothelial cells (Fig. 6), supporting the hypothesis that HOPE provides more protection for endothelial cells during reperfusion. Platelet aggregation after reperfusion leads to intrasinusoidal mechanical occlusion and exacerbates sinusoidal endothelial cell viability loss and microcirculatory blood flow disruption. Elevated liver enzyme levels after reperfusion illustrated the disturbance in sinusoidal microcirculation. Our experiments revealed that the HOPE group had significantly better liver enzyme (AST and LDH) levels after reperfusion (Fig. 3A,B).

### Hepatocyte effects

Current research suggests a key role of hepatocyte-released DAMPs during early reperfusion, with a steep increase during the first 4–6 h^17,32,33^. Reperfusion injury shifts from pure metabolic distress to a potentially lethal innate immune response^32^. HOPE triggers a unique decrease in DAMPs levels during early reperfusion of liver grafts obtained from donors after cardiac death^8,13,14,34^. In our experiment, DAMPs levels (TNF-α, interferon-γ, interleukin-1β, and interleukin-10) were significantly lower in the HOPE group than in the CS group (Fig. 7). Our results were similar to our previous findings and those of prior reports.

### Mitochondrial effects

Selective accumulation of the citric acid cycle has been recently shown as a universal signature of ischemia and is responsible for mitochondrial ROS production in all cell types during reperfusion^35^. Upon reperfusion, the accumulated succinate is rapidly reoxidized, driving extensive ROS generation by reverse electron transport at mitochondrial complex I^35,36^. HOPE prevents the initial mitochondrial ROS release and appears as a novel treatment that shifts anaerobic metabolism to aerobic metabolism under cold conditions, together with huge ATP reload. Our results were similar to our previous findings and those of prior reports (Fig. 3H).

HOPE-preserved liver grafts release potassium in the perfusate during MP and take up potassium upon reperfusion. This prevents acute hyperkalemia, which is frequently associated with severe post-reperfusion syndrome. HOPE is likely to impair optimal Na+/K+-ATPase function, facilitating passive potassium release. This underlines the potential role of ATP-dependent hepatic potassium uptake in HOPE-preserved livers^37^. In our experiment, at 60 min and the end of reperfusion, the HOPE group had significantly lower potassium levels than the CS group (Fig. 3F).

### Cellular defense effects

Low ROS levels are protective and may trigger the activation of numerous pathways (PKCe, SIRT1, Nrf-2, and HIF-1). These pathways increase antioxidant enzyme activation (glutathione synthase, heme oxygenase, catalase, glutathione, and manganese superoxide dismutase) and expression of angiogenic (erythropoietin) and survival proteins (mitogen-activated protein kinase)^33^. Furthermore, hypothermic MP may upregulate defense pathways via minor ROS release during cold perfusion, particularly in livers exposed to warm ischemia before perfusion.

In our study, the levels of TNF-α, a representative factor of DAMP, were significantly lower in the HOPE group and a hepatocyte-protective effect was observed (Fig. 7A). The AST and LDH levels remained significantly lower in the HOPE group during reperfusion (Fig. 3A,B), suggesting a hepatocyte-protective effect. During reperfusion, when the blood flow is reestablished, the damage caused during the ischemic period is aggravated by reoxygenation. This is initiated by the mitochondrial release of ROS due to an inhibited electron transport chain causing Kupffer cell activation, which releases proinflammatory cytokines (including TNF-α/interleukin-1β), recruits neutrophils, and induces adhesion molecule expression in sinusoidal endothelial cells. Activated neutrophils produce more ROS, perpetuating the inflammatory response that ultimately results in tissue damage and initiation of cell death programs^37–39^. Additionally, endogenous carbon monoxide is produced by heme metabolism in humans^40^. Heme oxygenase-1 is a rate-limiting enzyme for heme degradation and a stress protein induced by oxidative stress^41^. CO-Hb levels were lower in the HOPE group (Fig. 3G), suggesting less oxidative stress after reperfusion.

In addition, as compared to extracorporeal splitting, intracorporeal splitting has the following advantages: (1) shorter cold ischemia time; (2) better identification of the bile duct and vascular tissues, exact management of the liver section, and reduced incidence of section bleeding and bile leakage; (3) reduced rewarming injury; (4) ability to observe the blood supply and reflux in each liver segment after splitting, facilitating more rational allocation of donor vessels; and (5) complete hemostasis of the section during splitting, thereby reducing sectional bleeding during reperfusion in the recipients^42^. Therefore, application of extracorporeal splitting with MP to simulate intracorporeal splitting tends to take full advantage of the latter^43^. Mabrut et al. described the first two cases of liver transplantation with concurrent liver splitting and HOPE^44^. We have reported good results of splitting during MP^6^.

Here, we simulated and evaluated post-transplant reperfusion using an ERM. The livers were evaluated by reperfusion with oxygenated diluted autologous blood at 38 °C rather than after transplantation. We reported that the ERM can be useful for evaluating MP utility^19,20^. This isolated ERM can reduce the need for animal transplantation experiments and the technical bias associated with the actual transplantation operation, and facilitate observation of hemodynamic changes (perfusion area and vascular pressure resistance) during reperfusion. The ERM can be used to investigate events occurring in the early phases of reperfusion. Indeed, parameters of interest and biomarkers released during isolated liver perfusion tend to mainly reflect hepatocellular and endothelial injury and, to some degree, cholangiocyte injury, showing a pattern that fairly resembles our current knowledge of the sequence of events occurring in the early stages of IRI in animal experiments and clinical transplantation^45^.

### Study limitations

This study has some limitations. First, the present study involved 2 h assessment using an ERM. Hence, we need to conduct long-term assessment of the immune response, particularly in actual transplantation. However according to previous study, 2 h assessment after reperfusion was enough for evaluating the efficacy of HOPE in this model. Second, bile assessment was not performed in this study. Lastly, this study did not include larger split-liver grafts.

In conclusion, end-ischemic HOPE for split-liver transplantation can help recover graft function and reduce IRI. HOPE, using CMP-X04W, is safe and can improve graft function while attenuating liver injury due to preservation. This approach has shown significant advantages compared to the conventional CS and might expand this technology into recovery and amelioration of marginal grafts as split-liver grafts.

## Data Availability

The datasets generated during and/or analyzed during the current study are available from the corresponding author on reasonable request.
